# Scotch Physicians and Surgeons

**Published:** 1888-03-24

**Authors:** C. G. Furley


*(To be continued.)*

### Scotch Physicians and Surgeons.

By C. G. Furley.

*(Continued from p. 411.)*

### SIR ROBERT CHRISTISON.

Christison was of course on the academic side in the quarrel with the councillors. As a matter of scientific reason and aristocratic instinct alike, he disapproved of the interference of the respectable but unenlightened citizen in matters which he held to be beyond his ken. The descendant of the land and sea rovers could sympathise with many things, but not with the tradesman's spirit that preserved an impartial ignorance of the acquirements of the several candidates for a university chair, but voted for one or another at the bidding of "a lady that had given him a good job." Christison would not have said, as an English herald did when asked to tell the arms of a benefactor to a public institution, "Mr. —— had no arms; he was not a gentleman, but an alderman;" but he had something of the spirit of the herald in his soul. Thus he toiled and fought ceaselessly to have the privileges of the municipality in this respect taken away from them, and never rested till they were first abrogated, and finally, by the Universities Act of 1858, wholly withdrawn.

At the time Christison began his professional career little had been written on the subject of medical jurisprudence in England; and his first course of lectures was prepared from study of the medico-legal science of France, then far ahead of England in such studies. But this, though valuable in many respects, could give no instruction on questions involving the dicta of Scots law, and he had to reconstruct them on a more practical basis. Then, perceiving from a few translations that a vast mine of medico-legal facts lay concealed from view in the intricacies of the German language, he set to work to study that tongue. He commenced this formidable task in an original manner. After spending one day in studying the grammar, he began to read Schiller's "Thirty Years' War," and in fourteen days could read at the rate of ten pages an hour. This continuous study, enlarged as it was by frequent appearances as medical witness in criminal cases, resulted in a constantly-improving course of lectures, which attracted a constantly-increasing number of students; and this brought the reward he cared for most—that public recognition of the importance of the subject he taught, implied in its being raised from an optional to a compulsory subject for those who sought the degree of M.D. This was in 1833, just after Christison had been translated to the chair of Materia Medica; but the result was undeniably due to his labours. The subject of hygiene, as affecting either individuals or communities, was not then definitely recognised as a branch o medical jurisprudence; but Christison took it up, and even after he had left that part of medical science did much to give it the importance it now possesses. His address as President of the Public Health section of the Social Science Congress in 1863 was one of the most comprehensive that had then been given, and even now, when so much more has been learned of the subject it treats of, its scope and thoughtfulness command respect.

Although frequently called upon to exercise it, Christison disliked what he called "the trade of medical witness." The fashion of the lawyers of those days was to bully the medical witness, who was presumably unacquainted with courts of law, into some self-contradiction, and thus to discredit his testimony; they affected a disbelief in his veracity and impartiality, which seemed nothing less than a personal insult to a man of Christison's temperament. In fact, they did not want impartiality. In one case in which he was engaged, the lawyer on the side that called him complained that his evidence favoured the opposite side rather than that for which he was employed. It was useless to try to convince this gentleman that whatever his duty in the case might be, the doctor's was to find out the truth; but he was at last persuaded that it was useless and even stupid to invent lies which the other side would discover. Finally, however,

Christison was generally employed by the Crown, by which truth was more desired than startling evidence; and so he frequently came to stand as umpire between the prisoner and the prosecution. In this way he had to do with most of the remarkable poisoning cases of many years. Among others he was employed in that strange sad case of Madeline Smith, where a young girl was accused of poisoning a lover of whom she was tired, but who refused to give up his claims upon her. At the same time her parents were urging her to marry another suitor; and just when the complication became hopeless, the first—the clandestine—lover died suddenly from arsenic. There was no doubt of the cause of death; the only question was whether it was brought about by murder or suicide. Public opinion inclined strongly to the former theory; but public sympathy was averse from condemning a woman, young and ignorant, and maddened by the threats her lover had held over her, to death; and Madeline Smith escaped under the Scottish verdict of "Not proven."

In this case, as in others, the counsel on each side strove to induce the medical witnesses to go beyond their province, into the psychical and moral possibilities of the question, and Christison had to make it distinctly felt that anything beyond the matter of arsenical poisoning was no business of his, and that the inferences to be drawn from the prisoner's conduct before and after the crime did not belong to his functions.

To improve the careless way in which these inquiries were conducted at the time, Christison drew up, at the request of the Lord Advocate, a set of rules for future guidance. This is still in use, and it contains notes on certain points which the inexperienced are unlikely to note, and whose significance they mistake. For example: The slight differences that exist between injuries inflicted before death and after; and the apparent similarity between these and the pseudo-morbid appearances due to natural causes after death. Again, he drew attention to the necessity for observing every characteristic of wounds, especially wounds caused by cutting or puncture. Their seat, form, size, edges, direction, the organs traversed, foreign bodies found in or near them, the loss of blood indicated by them—all help to tell the weapon employed, the force necessary, the manner of striking, the relative positions of assailant or assailed, the occupation of the assailant, even the very person guilty; or if suicide is the only explanation.

The lack of clear and systematic information on the various branches of Medical Jurisprudence induced Christison to write that Manual of Toxicology by which his name is most widely known. "Christison on Poisons," though published in 1829, is still a standard work. His attention was drawn first to oxalic acid, which he investigated so thoroughly that nothing of importance connected with poisoning by that substance has since been added to our knowledge. He next took up arsenic, then opium, and so on till the material for the treatise accumulated. The most interesting part of this work for the general reader is the prefatory dissertation on poisoning in general, which is also the part of which the author himself thought most highly. His subsequent work on "Granular Degeneration of the Kidneys," published in 1839, after considerable study of the phenomena of Bright's disease, deals with a subject less clearly within his own province, and has not achieved as great a popularity as his earlier book.

In 1832 Christison was translated to the chair of Materia Medica, which he filled for forty-five years. While engaged in his professional work in connexion with this chair, his analyses included a large number of medicinal substances, but the poisons still retained their fascination for him. No one who ever heard him lecture on the subject will forget how he was wont to allude to a certain experiment with the ordeal bean of Old Calabar: "It was necessary in the course of my investigation of the action of Calabar bean to make what our friends the anti-vivisectionists would call a very cruel experiment. This experiment, gentlemen, I made on—myself." There can be little doubt that he liked the applause of his students that invariably greeted this announcement. Then he would narrate how, having taken one-eighth of a seed without feeling any evil effects, he doubled the dose, with the result of producing in fifteen minutes a giddiness, which increased, and was accompanied by a peculiar torpidity. Being thus satisfied that he had taken a dangerous dose of an energetic poison, he, with characteristic readiness of resource, swallowed the shaving water he had just been using, which promptly acted as an emetic. Enough, however, had already been absorbed into the blood to produce alarming symptoms—great disorder and weakness of the circulation, extreme muscular prostration, and difficulty of breathing—which lasted for several hours. Still, the point on which he sought assurance was settled—that the principal action of the poisonous principle of the bean (strychnine) was that of depressing, and in fatal cases paralysing the heart. Among other contributions to toxicology, Christison was the first to draw attention to digitaline, the active principle of foxglove. He also devoted some attention to hemlock and its principle, conine; coming to the conclusion that the tradition which assigned it as the instrument of Socrates' death was false.

